# CXCL12 and Its Isoforms: Different Roles in Pancreatic Cancer?

**DOI:** 10.1155/2019/9681698

**Published:** 2019-06-02

**Authors:** Alessandra Righetti, Matteo Giulietti, Berina Šabanović, Giulia Occhipinti, Giovanni Principato, Francesco Piva

**Affiliations:** Department of Specialistic Clinical and Odontostomatological Sciences, Polytechnic University of Marche, Ancona 60020, Italy

## Abstract

CXCL12 is a chemokine that acts through CXCR4 and ACKR3 receptors and plays a physiological role in embryogenesis and haematopoiesis. It has an important role also in tumor development, since it is released by stromal cells of tumor microenvironment and alters the behavior of cancer cells. Many studies investigated the roles of CXCL12 in order to understand if it has an anti- or protumor role. In particular, it seems to promote tumor invasion, proliferation, angiogenesis, epithelial to mesenchymal transition (EMT), and metastasis in pancreatic cancer. Nevertheless, some evidence shows opposite functions; therefore research on CXCL12 is still ongoing. These discrepancies could be due to the presence of at least six CXCL12 splicing isoforms, each with different roles. Interestingly, three out of six variants have the highest levels of expression in the pancreas. Here, we report the current knowledge about the functions of this chemokine and then focus on pancreatic cancer. Moreover, we discuss the methods applied in recent studies in order to understand if they took into account the existence of the CXCL12 isoforms.

## 1. Introduction

Pancreatic ductal adenocarcinoma (PDAC) is one of the most lethal gastrointestinal tumors; indeed, it is characterized by poor prognosis and the survival rate is only 8%. Because of asymptomatic clinical course, patients at the moment of diagnosis already present advanced or spread diseased stage. In particular, more than 80% of patients have unresectable carcinomas at the moment of diagnosis. The mutation of oncogene K-Ras, an earliest genetic event, is the first factor which promotes the progression of ductal epithelial cells from a normal state to a neoplastic intraepithelial conformation (pancreatic intraepithelial neoplasia, PanIN) [1–3]. As a consequence of mutations in this protein, several downstream processes are activated, such as proliferation, metabolic reprogramming, antiapoptosis, evasion of the immune response, and remodeling of the microenvironment. PDAC cancer cells are composed of different subpopulations, such as epithelial cancer cells, CD24^+^/CD44^+^ cancer stem cells, CD133^+^ cancer stem cells, and mesenchymal cancer cells; furthermore, each cell population is genetically heterogeneous. However, to understand the tumor onset, behavior, and drug resistance, it is important to study also the stromal component of PDAC (cancer microenvironment). This component consists of several cell types: cancer‐associated fibroblasts (CAFs), T cells, pancreatic stellate cells (PSCs), macrophages, endothelial cells, and others [4, 5].

Cancer cells and surrounding microenvironment exchange signals with each other by releasing signaling molecules [6]. In particular, PSCs produce extracellular matrix molecules (i.e., laminin, fibronectin, and periostin) [7–10] while macrophages release matrix metalloproteinases (e.g., MMP-9) [11]. Both tumor and stromal cells release growth factors, including FGF, EGF, VEGF, HGF, and TGF-*β* and the inflammatory messenger IL-6. Instead, COX-2 is released by macrophages [11–13]. The chemokines CXCL12, CCL2, and CCL22 are produced by CAFs and macrophages. In particular, CXCL12 is mainly released by CAFs, which represent the 50% of tumor stroma [8]. All these released factors promote the activation of numerous signaling pathways, crucially linked to PDAC development [14]. Among inflammatory cytokines, the CXCL12 chemokine plays an important role in tumor-stroma communication. In PDAC microenvironment, the CAFs activation is induced by molecules released by cancer cells, in particular, IL-6, TGF-*β*, IL10, PDGF, and FGF [12, 13]. The activated CAFs release the CXCL12 chemokine, which binds to its two receptors, CXCR4 and ACKR3, highly expressed on the cancer cells surface [8, 15, 16]. This binding allows the activation of numerous signaling pathways in pancreatic cancer cells, such as phospholipase C, MAPK, and PI3K-Akt-mTOR, as well as JAK/STAT pathways. The activation of one or more of these pathways supports the tumor growth and invasion, promotes resistance to drug therapy, provides possible niches for the metastasis development, and protects tumor cells from the host's immune system [17, 18].

Regarding the latter, it is known that CXCL12 exerts a predominant immunosuppression effect by sequestering CD8^+^ T cells and preventing them from attacking the cancer cells [19]. Moreover, by depleting fibroblast activation protein (FAP)-expressing CAFs, it was possible to attain immune control of the PDAC development and to restore the antitumor effects of anti-CTLA-4 and anti-PD-L1 immune checkpoint antagonists [20–22].

## 2. Chemokines

**Figure 1:**
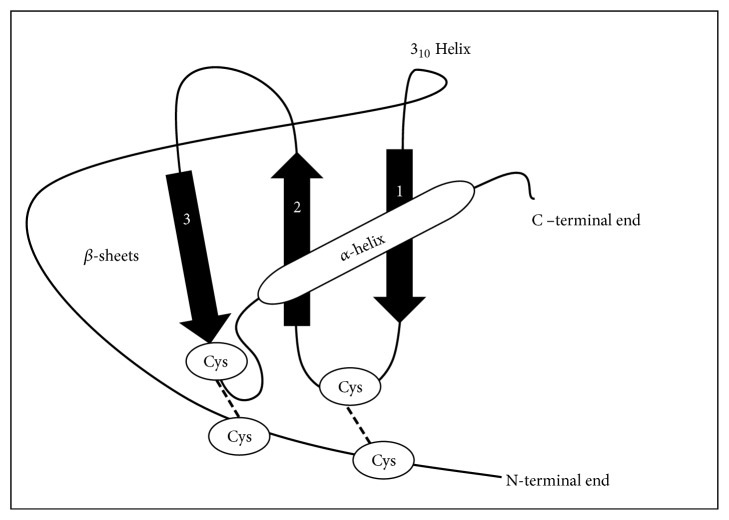
CXC chemokine structure. Chemokine presents three antiparallel *β*-sheets and a C-terminal *α*-helix; this tertiary structure is owing to the presence, starting from N-terminal region, of an “N-loop”, a single 3_10_ helix, and then the 30s and 40s loops. The two cysteine residues at the N-terminal end allow the formation of disulphide bonds which are fundamental to the chemokine structure and receptor interaction.

Chemokines are a family of low molecular weight proteins (8-14 kDa) involved in the immune system's response. They activate leukocytes and direct their migration from the circulation to an inflammation site. Among chemokines there is high sequence homology (about 20-50%); indeed their tertiary structure stability is due to conserved amino acids, like cysteines, that form the characteristic chemokine “Greek key” (three antiparallel *β*-pleated sheets are overlaid by a C-terminal *α*-helix) holding protein structure through two disulphide bonds [23] (Figure 1). Chemokines are synthesized from a propeptide, consisting of different amino acids number based on the protein, which is subsequently removed during the cell secretion of the mature protein. Most chemokines are equipped with four residues of cysteine, two of which are located at the N-terminal end, one in the middle, and one near the C-terminal end. The portion preceding the first *β*-sheet consists of about 20 residues at the N-terminal end, the motif with the conserved cysteine (CC, CXC, CX3C, or C), an “N-loop”, and a single 3_10_ helix. The first disulphide bond occurs between the first conserved cysteine residue and the 30s loop, which is located between the first and the second *β*-sheet while the second disulphide bond is between the second conserved cysteine residue and the 40s loop, located in the third *β*-sheet. These disulphide bonds give the structure to the chemokines and the ability to integrate with the receptor [24, 25].

About 45-50 chemokines, all structurally similar, have been identified in humans. They are classified into 4 subfamilies based on the localization of the cysteine residue in the NH2-terminal region. The class of CXC chemokines (*α*-chemokine) comprises chemokines with two cysteine residues separated by one amino acid. In mammals, 17 different chemokine CXCs were identified; these, in turn, can be divided into two categories: the “ELR-positive” chemokines, with the specific amino acid motif (the ELR motif Glu-Leu-Arg), immediately before the first cysteine, and the “ELR-negative” chemokines which do not present this sequence. In neutrophils, for the interaction between ligand and receptor, this three-amino-acid sequence is very important and is highly conserved in all members of the CXC chemokine family. The subfamily of CC chemokines (*β*-chemokine) is characterized by the presence of two adjacent conserved cysteine residues; they are also called the CC chemokine ligands (CCLs). Twenty-seven members of this group have been identified. Most of them can contain four cysteine residues; others can also have six cysteines. CC chemokines trigger the movement of monocytes, natural killer cells, and dendritic cells. In the class of C chemokines (*γ*-chemokines) only two chemokines have been described: XCL1 (lymphotactin-*α*) and XCL2 (lymphotactin-*β*). They are different from all other chemokines because they have only one NH2-terminal cysteine residue. The CX3C chemokine (*δ*-chemokine) subfamily presents two cysteine residues at the NH2-terminal that are separated by three amino acids. Only one chemokine has been discovered, that is, fractalkine (or CX3CL1). It is both released by and bound to the cell that expresses it and acts as both a chemoattractant and a cell adhesion molecule [24–30].

### 2.1. Chemokine Functional Roles

Chemokines and their receptors play important physiological roles in the human organism. Through a concentration gradient, they act as chemoattractant factor driving the cellular migration. Based on their constitutive or inducible production, they are classified into homeostatic and inflammatory chemokines, respectively. The homeostatic chemokines are produced in the thymus and lymphoid tissues and do not need to be stimulated by external stimuli. Some chemokines control the immune surveillance process directing the leukocyte homing; others play a role in embryogenesis, haematopoiesis, and neurogenesis and promote angiogenesis [31]. Homeostatic chemokines are CCL14, CCL19, CCL20, CCL21, CCL25, CCL27, CXCL12, and CXCL13. The inflammatory chemokines are released by many different cell types and drive the cells of both adaptive and innate immune system. Inflammatory chemokines are produced in high concentrations during infection or injury and they act as a chemoattractant for leukocytes, recruiting monocytes, neutrophils, and other effector cells from the blood to sites of infection or tissue damage. Their production is stimulated by proinflammatory cytokines like interleukin-1. Typical inflammatory chemokines include CCL2, CCL3, and CCL5; CXCL1, CXCL2, and CXCL8 [29].

### 2.2. Chemokine Receptors

Chemokine receptors are mainly anchored on the leukocyte surface and, based on their mechanism of action, are divided into two groups: G-protein-coupled receptors (GPCRs), which activate signaling through G proteins, and atypical receptors, acting through the binding with *β*-arrestin [24]. About 18 chemokine receptors have been identified and are characterized by 7-transmembrane domains. They are classified into the CXCR, CCR, CR, and CX3CR groups based on the respective chemokine family they can bind. However, the interaction between a chemokine and its receptor is not completely exclusive; indeed, each receptor can recognize more than one chemokine type, and a chemokine can bind multiple receptors [29].

Approximately 350 hydrophilic and hydrophobic amino acids constitute these receptors. Going from extracellular to intracellular environment, there are (i) a short N-terminal region that allows the specificity of ligand binding, (ii) seven-transmembrane domains that lead to the formation of 3 extracellular and 3 intracellular loops, and (iii) a C-terminal region suitable for receptor regulation and for G proteins binding that triggers the intracellular signaling after receptor activation [32]. In addition to the C-terminal portion, G proteins can also bind to the receptor through the third intracellular loop [32]. The interaction between chemokines and their receptors induces a receptor conformational change, with consequent signal transduction and, in the end, the cellular responses [24], such as chemotaxis, immune cell migration, and inflammation but also tumor initiation, promotion, and progression [29].

## 3. CXCL12

CXCL12 (C-X-C motif chemokine ligand 12) is one of the most studied chemokine proteins. It has a key role in both physiological and pathological conditions. Originally, CXCL12 was identified as a pre-B cell growth factor (PBGF), which plays an important role in homeostatic processes. Subsequently, it has actually been discovered that PBGF is constitutively expressed by bone marrow stromal cells, so it has been called factor 1 derived from stromal cells (SDF1) [27, 31]. This homeostatic chemokine plays a constitutive role in physiological processes such as embryogenesis, haematopoiesis, angiogenesis [31, 33, 34], development of cardiovascular and nervous systems [35], regulation of different cell functions like differentiation, distribution, activation, immune synapse formation, effector function, proliferation, and survival in the immune system [33]. In contrast to all these physiological functions, it also plays an inflammatory role in many diseases. It is involved in different pathological processes, such as HIV infection, neovascularization, chronic inflammatory disorders, tumor growth, distant metastasis formation, and chemoresistance [28, 31, 33, 36–38]. In cancer, the binding between CXCL12 and its receptors causes different pathways activation, that, through cancer cells, migration, angiogenesis, and epithelial to mesenchymal transition (EMT) [39], are involved in tumor initiation and progression [36, 40].

Contrarily to common CXC chemokines, whose genes are found on chromosome 4q21, the gene that encodes the protein CXCL12 is found on chromosome 10q11 [28]. Although seven different splicing variants of CXCL12 chemokine have been discovered (*α*, *β*, *γ*, *δ*, *ε*, *θ*, and the predicted isoform iso7), the functional roles of each one are still unknown. Until now, most studies have focused mainly only on three isoforms, i.e., *α*, *β*, and *γ*. Out of four exons in total, the first three are shared with all other splicing variants of CXCL12, but each one differs from the others in the terminal region.

**Figure 2:**
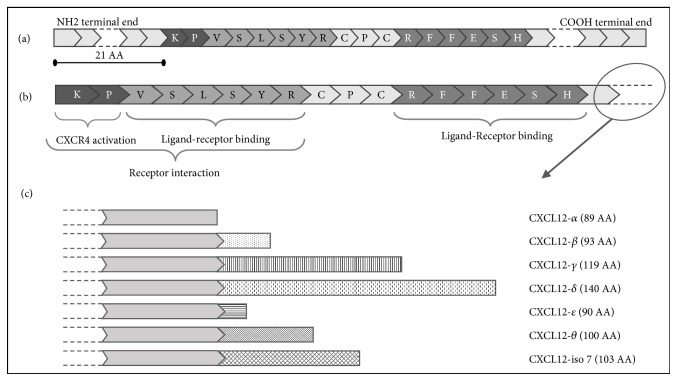
CXCL12 sequence. (a) The CXCL12 immature form, the propeptide, which includes the 21 amino acids at the N-terminal end, that will be removed. (b) The mature CXCL12 form has undergone a proteolytic cut of 21 amino acids at the N-terminal end. The first 8 amino acids of the mature CXCL12 allow the receptor interaction; in particular, the first two, lysine and proline, activate the CXCR4 receptor while the other six are used for the receptor binding. Also, the “RFFESH” sequence allows the ligand-receptor binding. (c) Representations of all CXCL12 isoforms are reported. They all have the same starting sequence, but each one differs from the others in the terminal region length.

In order to be biologically active, CXCL12, initially secreted as propeptide, is subjected to the proteolytic removal of 21 amino acids present at NH2 terminal end (Figure 2). This monomeric mature form, which has undergone cutting, is unstable at blood level and tends to glycosaminoglycans (GAGs) binding, escaping proteolytic degradation [41, 42]. Three parallel *β*-strands and an overlying *α*-helix constitute the ordered structure present among the disordered N- and C-terminal ends. In the mature form of CXCL12, the first 8 amino acids (AA) at the N-terminal play an important role in the interaction with the receptor; in particular, the first two AAs (Lys-1 and Pro-2) activate CXCR4, while the other six allow ligand-receptor binding [28, 37]. Another element exists in the CXCL12-CXCR4 interaction: the RFFESH sequence. This motif is involved in ligand-receptor binding and, thanks to the structural changes, it allows the N-terminal AA activation of the receptor (Figure 2) [27]. In the stabilization of the receptor binding (between the organized region and disorganized C-terminal one), glycosaminoglycans (GAGs) such as heparin and heparan sulphate play an important role. Thanks to their negative charge, these long polymers of disaccharide units form an extracellular matrix that attracts positively charged chemokines towards itself. This chemokine-GAG interaction is essential for the chemokine gradient generation. The binding to the GAGs protects CXCL12 from NH2-terminal truncation and inactivation [27, 28, 37].

### 3.1. Expression

CXCL12 is expressed in various human tissues (liver, pancreas, spleen, and heart) by different cells, like stromal cells, fibroblasts, and epithelial and dendritic cells [36, 43–45]. Only blood cells do not seem to express CXCL12. Furthermore, CXCL12 can be secreted in tumor microenvironment by carcinoma-associated fibroblasts and mesenchymal cancer stem cells [37, 46]. The expression and activity of CXCL12 are regulated by three different factors: hypoxia, ACKR3 scavenging, and posttranslational modifications. Hypoxia is a characteristic component of inflammatory stages, that, through its tissue mediator HIF-1 (Hypoxia-Inducible Factor-1), has been shown to induce CXCL12 expression and secretion by fibroblastic and endothelial cells. Thanks to the presence of a HIF-Response Element (HRE) on the CXCL12 promoter, HIF-1 is able to regulate the promoter's transcription activity [47, 48]. The second regulatory factor involves the atypical receptor ACKR3. It can act as a scavenger, influences the chemokines gradient, and decreases inflammation. Indeed, it sequesters and internalizes CXCL12 to allow the activation of downstream pathways or the lysosome ligand degradation [49]. Regarding the posttranslational modifications, which alter the function of CXCL12, they involve both chemical and enzymatic modifications, including NH2-terminal truncation, COOH-terminal truncation, citrullination, and nitration. The NH2-terminal truncation is implemented by serine proteases, in particular, the transmembrane serine protease dipeptidyl peptidases IV (DDP4) and the intracellular serine protease dipeptidyl peptidases VIII (DPP8). DPP4, also called CD26, splits proteins containing an Ala or Pro residuals in the penultimate position of their amino acid sequence. The DPP4 cleavage of CXCL12 induces a loss of calcium-dependent signaling and chemotaxis. These changes lead to a decrease in the binding ability of GAGs (especially heparin) and CXCR4. Moreover, DPP4-truncated CXCL12 is inactivated but still able to bind the CXCR4 receptor, so it acts as an antagonist [31, 50]. Being an intracellular protease, DPP8, differently from DPP4, can cleave CXCL12 only after ligand internalization or DPP8 release after cell lysis.

Regarding the COOH-terminal truncation, the enzymes involved in this modification are the secreted carboxypeptidase N, the plasma membrane carboxypeptidase M, and the lysosomal Cathepsin X. Due to the presence of the lysine at the C-terminal end, the carboxypeptidase M and N degrade only the *α*-isoform [37, 42]. CXCL12 degradation occurs in both blood and tissues and does not inactivate CXCL12 but halves its activity and receptor binding affinity. This causes lower receptor binding capability, chemotaxis, cell proliferation, GAGs binding, and a greater predisposition to NH2-terminal truncation. Also, Cathepsin X decreases CXCL12 activity.

The enzyme implicated in the citrullination is the peptidylarginine deiminase (PAD) and involves the hydrolysis of the imine group of Arg into the ketone group, resulting in citrulline (Cit) formation. This switch from Arg to Cit causes an alteration of protein structure and subsequent modification of its interaction with other proteins. The effects on CXCL12 activity are inhibition of receptor binding, signal transduction, and chemotaxis. The CXCL12 citrullination has a greater impact on decreasing CXCR4 activity than ACKR3.

The nitration process takes place by the chemical factor peroxynitrite and leads to the nitration of the residues of Tyr and Trp with the formation of nitrotyrosine. Tyrosine nitration can either increase or decrease protein activity or have no effect on it. The CXCL12 nitration can be induced chemically or it can occur naturally. This nitration, assessed by in vitro studies with lymphocyte and monocyte, results in reduced intracellular calcium mobilization, IP3 accumulation, and ERK1/2 phosphorylation, with the consequent decrease in chemotaxis. It also reduces cellular signaling and migration [27, 31].

Various cell and tissue types secrete CXCL12 whose expression is regulated by enzymatic or lysosomal degradation. CXCL12 has a rather short half-life in the bloodstream, about 30 minutes, due to processes such as degradation by proteases, binding to GAGs, and following sequestration. The above-mentioned processes permit gradient formation in various compartments [34, 42, 46]. It has been demonstrated that enzymes responsible for proteolytic degradation are metalloproteinases (DDP4 and MMP2) and leukocyte elastase (Cathepsin G). Still, there are no data about their roles in physiological in vivo processes [43, 51–54]. Nevertheless, at the cellular level, CXCL12 is digested by lysosome after being internalized by ACKR3 receptor [55, 56].

### 3.2. Dimerization of CXCL12 Receptors

CXCR4 and ACKR3 can form homo or heterodimers on the cell membrane. Depending on whether CXCL12 binds to its monomeric or dimeric (homo-ether dimer) receptors, different signaling pathways may be activated [33]. In particular, (i) the CXCR4 monomer signaling is preferably mediated by the G proteins which activate the MAPK/PI3K/Akt pathways; (ii) the CXCR4 homodimerization, induced by CXCL12, involves the activation of the JAK/STAT pathway, but it is not known if G proteins or *β*-arrestin are involved; (iii) the ACKR3 monomer acts as a CXCL12 scavenger and activates ERK 1/2 signaling via *β*-arrestin; (iv) the CXCR4/ACKR3 heterodimer formation shifts the signaling from G proteins to *β*-arrestin. By GRK-dependent phosphorylation, *β*-arrestin signaling may cause the CXCR4 internalization, the CXCL12 scavenging, and/or the activation of the ERK1/2 pathway resulting in cell survival increase.

CXCR4 is internalized and degraded only after CXCL12 binding; instead, ACKR3 is continuously internalized independently of the ligand binding [41, 55] and it is not degraded [41].

### 3.3. CXCL12 Interactions

Through the interactions with two different CXC chemokine bind receptors, CXCR4 (CD184) and ACKR3 (CXCR7), and through the glycosaminoglycan (GAGs) binding, the chemokine CXCL12 plays a role in physiological and pathological conditions.

#### 3.3.1. CXCR4

CXCR4, also known as cluster of differentiation 184 (CD184), is characterized by seven-transmembrane domains, usually categorized as G-protein-coupled receptor (GPCR). Originally, CXCR4 was identified as leukocyte-derived seven-transmembrane domain receptor (LESTR) for its role as a cofactor in human immunodeficiency virus (HIV) cell entry. Subsequently, its role as the receptor of CXCL12 ligand was discovered [27]. Regarding the extracellular structure, CXCR4 has a different organization compared to normal GPCR receptors. Indeed, this receptor, at the end of helix VII, has two helical turns longer than the other receptors. This extension allows the formation of a disulphide bond between Cys274 and Cys2, and together with the extracellular loop 2 (ECL2, Cys186) and the extracellular end of alpha helix III (Cys109) is essential for binding with CXCL12 [28, 34]. In the last ten years, the interest in this receptor is exponentially increased because many cell types express it, including the cancer cells. In particular, it is expressed in leukocytes, lymphocytes, epithelial and hematopoietic progenitor stem cells, cells of lymphoid organs like the bone marrow, thymus and lymph nodes, lung, small intestine, and nonhematopoietic cells, such as endothelial, epithelial, and stromal cells (fibroblasts). The gene encoding this receptor is located on chromosome 2. CXCR4 has two alternative isoforms: CXCR4-A and CXCR4-B. The CXCR4-B isoform is more expressed and undergoes a splicing process, while the CXCR4-A isoform does not undergo splicing, differs in 5 amino acids, and is four amino acids longer at the NH2 terminal. Functionally, they are both active [27, 31]. Regarding the regulation of CXCR4 expression, it is known that the Nuclear Respiratory Factor 1 (NRF-1) and HIF-1 increase its transcription levels, while the transcriptional suppressor Ying Yang 1 (YY1) inhibits its expression. Numerous molecules induce transcription of CXCR4, such as growth factors and cytokines (BFGF, VEGF, interleukin, and TGF-*β*), as well as second messengers: calcium and cyclic AMP [28, 34, 57].

#### 3.3.2. CXCL12/CXCR4 Pathway

The CXCR4-mediated signaling occurs mainly by G proteins binding. The CXCL12 binding to CXCR4 extracellular portions induces a conformational change of the receptor tertiary structure that triggers the heterotrimeric G-protein (G_*αβγ*_) activation (linked to the DRYLAIV sequence present in the second intracellular loop of CXCR4) and, in turn, by converting GDP in GTP dissociates into *α*-subunit (G_*α*_) and *βγ*-complex (G_*βγ*_). After CXCL12/CXCR4 binding, the receptor is internalized and degraded by lysosomes [41]. The *α* subunits can be G_*α*i_, G_*α*q_, and G_*α*12/13_. Depending on subunit binding, CXCR4 can activate different signaling pathways, such as phospholipase C, MAPK, and PI3K-Akt-mTOR. The activation of one or more of these pathways results in cellular migration, proliferation, activation of adhesion molecules, and chemotaxis. In tumor, it is correlated with progression, survival, angiogenesis, and metastasis development [27, 34, 38].

CXCR4 also seems to trigger signaling through JAK2/3 binding; in particular, some authors think that JAK2/3 cause intracellular calcium mobilization and chemotaxis, through the kinases transphosphorylation and subsequent CXCR4 phosphorylation, resulting in STAT 1/2/3/5 recruitment and activation. Other authors, based on models of JAK-deficient cell lines, claim that CXCR4 does not use JAK/STAT to activate the downstream pathways [27, 34]. Finally, this receptor can also bind to *β*-arrestin and induce activation of the p38 MAPK pathway [27, 31].

#### 3.3.3. ACKR3

The second receptor for CXCL12 is the “atypical” chemokine receptor ACKR3, so called because of its different transduction activation method. Indeed, the activation of ACKR3 excludes the common G-protein signaling and occurs through *β*-arrestin binding. In the past, this receptor was named RDC1; subsequently, the orthologue GNR1 cloned from leukemic pre-B cells was wrongly identified as a polypeptide intestinal receptor. A few years later, RDC1 was classified as calcitonin gene-related peptide (CGRP). Consequently, with the discovery of binding to CXCL12, RDC1 was renamed as CXCR7 and then ACKR3 [58, 59].

Compared to the CXCR4 receptor, CXCL12 appears to bind to the ACKR3 receptor with a 10 times greater binding affinity (apparent *K*_*D*_ = 0.4 ± 0.1 nM for ACKR3, apparent *K*_*D*_ = 3.6 ± 1.6 nM for CXCR4) [60, 61]. In addition to CXCL12, ACKR3 can also recognize the chemokine CXCL11. In human, ACKR3 gene is located on chromosome 2 and it includes a different amino acid sequence (DRYLSIT) from the usual sequence of chemokine receptors (DRYLAIV). ACKR3 is expressed by different cell types, such as hematopoietic cells, activated and vascular endothelial cells, fetal hepatocytes, placenta cells, neuronal progenitor cells, and endothelial cells of tumor tissues. In particular, this receptor is highly expressed on the cell surface of T lymphocytes, during chemotaxis processes mediated by CXCL12. Its expression is associated with the ability of B cells to differentiate into plasma cells following activation [27, 28, 31]. This mechanism translates into several biological aspects such as regulation of the immune response and migration of T cells, stem cells, and neural progenitor cells.

There are contrasting opinions about the specific role of ACKR3 [33]. While some authors think that this receptor has a protumor role, since it induces cell migration and proliferation [55], others recognize an antitumor role (decoy role) of ACKR3 since it can prevent CXCR4-ligand binding by scavenging CXCL12 [56].

#### 3.3.4. CXCL12/ACKR3 Pathway

There is not much data on the CXCL12/ACKR3 pathway. It would seem that by *β*-arrestin binding, ACKR3 can induce the activation of Akt and its MAP kinases ERK1 and ERK2. Despite the lack of calcium mobilization, following the receptor activation, the *β*-arrestin pathway is activated and CXCL12 scavenger is obtained. Moreover, the signaling mediated by ACKR3, in addition to the CXCL12 sequestration from the microenvironment, promotes cell migration, survival, and adhesion [27, 28, 31, 58, 59]. Unlike CXCR4, this receptor, following its CXCL12-binding internalization, is not degraded but it is recycled on the plasma membrane [41].

#### 3.3.5. GAGs

The CXCL12 chemokine carries out its functions not only by binding to chemokine receptors but also by interacting with glycosaminoglycan (GAGs), such as heparin and heparan sulphate, which either are attached to the proteins of the cell surface or form the extracellular matrix itself. The negative charges of GAGs interact with the positive charges of the CXCL12 allowing the chemokine gradient formation [27, 31, 34].

## 4. CXCL12 Isoforms

**Figure 3:**
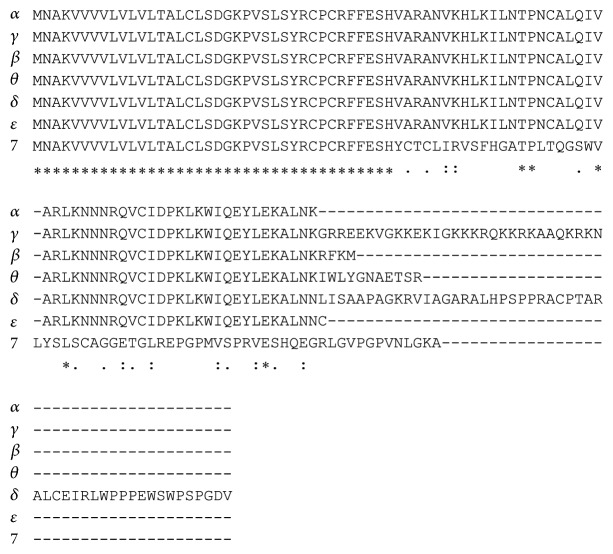
Protein multialignment of all CXCL12 isoforms.

Until now, six isoforms of CXCL12 have been discovered: *α*-isoform, *β*-isoform, *γ*-isoform, *δ*-isoform, *ε*-isoform, and *θ*-isoform; and the isoform-7 still remains predicted. The CXCL12 is the only CXC chemokine with alternative splicing variants and it is also the only one to be regulated and processed more at the posttranslational level than by transcriptional mechanisms. All seven isoforms share the first 3 exons (1-68 AA) and differ in length for the last exon. The amino acid sequence of this exon confers the specific length of each isoform: 89 AAs for *α*-isoform, 93 AAs for *β*-isoform, 119 AAs for *γ*-isoform, 140 AAs for *δ*-isoform, 90 AAs for *ε*-isoform, and 100 AAs for *θ*- isoform [27, 59] (Table 1, Figures 2 and 3).

Our protein alignment (Figure 3) shows that *α*, *β*, and *ε* isoforms differ from each other in a few AAs. Γ and *δ* isoforms are the longest; instead, isoform 7 is quite different from all the others.

It is known that the *α*, *β*, and *γ* isoforms differ in binding affinity with the extracellular matrix (ECM); in particular, *γ*-isoform has a greater affinity than *β*-isoform, which, in turn, is greater than *α*-isoform. Due to the binding with the extracellular matrix, CXCL12 is protected from cell degradation process and consequently has a slower tissues release. This mechanism leads to the formation of specific gradients based on the different affinities of isoforms ECM binding [37, 62].

**Figure 4:**
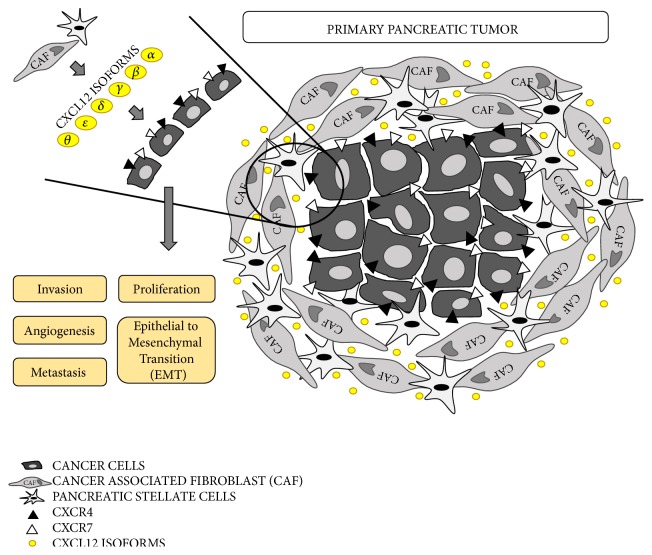
Primary pancreatic tumor microenvironment. PDAC stromal cells, like cancer-associated fibroblasts (CAFs) and cancer stellate cells, release the different CXCL12 isoforms in the tumor microenvironment. Cancer cells, which expressed the CXCL12-receptor on their surface, can bind the ligand. This ligand-receptor binding can cause tumor invasion, cellular proliferation, angiogenesis, epithelial to mesenchymal transition (EMT), and metastasis.

Each variant seems to have different expression and function [59], but we do not know with certainty their specific involvement in physiological processes [43, 63]. They also appear to be involved in tumor development processes such as apoptosis, metabolism, and development of metastases (Figure 4) [33].

### 4.1. CXCL12 *α*-Isoform

Among the variants of CXCL12, the *α*-isoform is the most studied. This variant is not present in the blood due to enzymatic degradation; instead, it is highly expressed in tissues, more in adult than in fetal ones [27, 37]. In particular, it is expressed in bone marrow, pancreas, liver, lungs, spleen, heart, lymph nodes, and thymus and has also been found in skin, small intestine, and neurons [34, 38]. Its amino terminal region makes it a specific ligand for CXCR4 and ACKR3 for the promotion of angiogenesis [28]. CXCL12-*α* can induce, in vitro, an increase in the survival rate of hematopoietic progenitor cells. Depending on the tissue expressing it, this isoform is able to manage hematopoietic stem cells in the bone marrow, to guide germinal cells during development, and to induce neurostimulation of the central nervous system. In breast cancer, low expression of CXCL12-*α* corresponded with worse metastasis-free survival [46].

### 4.2. CXCL12 *β*-Isoform

The *β*-isoform, despite functional similarities with the *α*-isoform, is particularly correlated with the vascular system. Thanks to differences in its fourth exon, this isoform is not degraded by blood carboxypeptidase N. Indeed, the sequence of this variant includes five additional residues at the C-terminal region, which contain one motif for HS (heparan sulphate) binding [64]. Like the *α*-isoform, it acts as a specific ligand for CXCR4 and ACKR3. It is highly expressed in vascularized organs like spleen, liver, bone marrow, pancreas, and kidney [37], in the endothelial cells of brain microvessels [38] and also in fetal tissues like liver and lung [27]. Unlike CXCL12-*α*, CXCL12-*β* promotes angiogenesis, as observed in vitro by the endothelial tube formation assay [65]. In bladder cancer, the mRNA levels of CXCL12-*β* are associated with poor prognosis and are potential predictor of metastasis and future recurrence [66]. In breast cancer, low levels of CXCL12-*β* correlated with worse metastasis-free survival and recurrence-free survival [46].

### 4.3. CXCL12 *γ*-Isoform

Thanks to its C-terminal end binding site, this isoform is the variant with the highest GAGs binding affinity and it is able to escape inactivation by proteolytic enzymes. CXCL12-*γ* is not expressed in fetal tissues but highly expressed and active in less vascularized organs, like heart and brain [27, 37, 59]. Once secreted, it binds immediately to the cell surface, reducing its presence as a free form [28]. Functionally, it is able to induce a weak in vitro chemotaxis, while in vivo it is the most active isoform which stimulates chemotaxis [27]. CXCL12-*γ*, thanks to its stable binding interaction, is characterized by a greater long-term effect than the *α*-isoform; indeed, in mice injected with both isoforms, it produces an inflammatory reaction 5 times longer [37]. As demonstrated in vitro by the endothelial tube formation assay, CXCL12-*γ* drives angiogenesis similarly to CXCL12-*β* [65]. In colorectal cancer, CXCL12-*γ* is positively associated with tumor size [44]. In prostate cancer, CXCL12-*γ* plays a key role in induction of cancer stem cell and neuroendocrine phenotypes, which are known to promote tumor growth, metastasis, chemoresistance, and the progression to the metastatic castration resistant prostate cancer [67]. In breast cancer, higher levels of CXCL12-*γ* correlate with improved metastasis-free survival and recurrence-free survival [46]. Unlike the other isoforms, CXCL12-*γ* significantly increased the breast cancer metastatic tropism for bone marrow [65].

### 4.4. The Other CXCL12 Isoforms

For about ten years, three additional isoforms of CXCL12 have been identified but their specific role has not yet been established. The highest levels of expression of each variant were found in the pancreas [27, 63]. The *δ*-isoform is also expressed in the liver, spleen, and lungs [37], while the isoforms *ε* and *θ* have also been found in the heart, kidneys, and liver [63]. In breast cancer, higher expression of CXCL12-*δ* correlates with better overall survival [46].

## 5. CXCL12 and Pancreatic Cancer

Numerous studies are trying to shed light upon the tumor roles of CXCL12, such as its effects at cellular levels and interactions with CXCR4 and ACKR3 receptors [68, 69]. The CXCL12/CXCR4 axis seems to play an important role in the processes of invasion, proliferation, migration, metastasis, and angiogenesis in pancreatic cancer (Figure 4) [17, 18].

Indeed, both pancreatic cancer cells and tissues highly expressed CXCR4 and ACKR3 receptors on their surface, which are activated by CAFs-released CXCL12 chemokine [70, 71]. In particular, according to immunohistochemistry data, 56.7% of pancreatic cancer tissues, 50.0% of para-cancerous tissues, and 53.3% of pancreas surrounding lymph nodes express CXCR4 compared to 18,3% of the normal pancreatic tissues [72]. Another study reported a positive CXCR4 expression in 80% of cancerous tissues, 70% of para-cancerous tissues, and 73.3% of lymph nodes compared to 26.7% of the normal pancreata [68]. Moreover, 73% of human pancreatic cancer tissues express both CXCR4 and ACKR3 [73]. Interestingly, pancreatic stellate cells isolated from pancreatic cancer tissues do not express CXCR4 [74, 75].

Regarding CXCL12 expression in normal pancreas, only ductal epithelial cells expressed CXCL12, whereas acinar and endocrine cells do not express it [71, 76]. In pancreatic cancer, the CXCL12-*α* isoform was moderately or strongly expressed at the protein level in primary pancreatic stellate cells isolated from PDAC tissues [74]. Moreover, ELISA assay revealed release of CXCL12-*α* from fibroblasts but not from pancreatic cancer cells [17]. However, in this paper, the “*α*-isoform” term is reported only in the title and keywords, but since the product identifier of the used R&D ELISA kit is not reported and R&D ELISA kits exist for total CXCL12 or for both *α* and *β* simultaneously, probably referring to the “*α*-isoform” is inaccurate.

In pancreatic cancer tissues, the expression of CXCL12-*α* isoform, assessed by western blot, was higher than adjacent tissues [77]. In addition, as seen by immunohistochemistry, 45.3% of PDAC tissues expressed CXCL12 protein and it was correlated with histological grades of disease severity [68]. On the contrary, there is other evidence that CXCL12 protein was frequently expressed in normal tissues (56.7%), in para-cancerous diseases tissues (46.7%), and in pancreas surrounding lymph nodes (50%); instead, only 13.3% of tumor tissues expressed it. However, it is not specified to which isoforms authors refer in these papers [78]. Regarding the last two works, we assume that data referred to the total CXCL12 but we cannot be sure since the product identifiers of the used antibodies are not reported in the Methods section.

Also in pancreatic cancer it has been shown that the interaction between tumor and surrounding stroma, mediated by the CXCL12/CXCR4 axis, influences the tumor growth and its aggressiveness. In particular, by administering the culture medium of pancreatic stellate cells (PSCs) to AsPC-1, BxPC3, and SW1990 cancer cells, an increase in proliferation, migration, and invasion due to activation of CXCL12-*α*/CXCR4 axis was observed [75]. In another study, it was reported that pancreatic stellate cells, which produced CXCL12-*α* chemokine, cause an increase in tumor growth [74].

Interestingly, chemoresistance was observed in pancreatic cancer cells treated with CXCL12-*α* and, subsequently, with mTOR-targeted therapies or gemcitabine. For example, it was shown that the activation of CXCR4 by CXCL12-*α* isoform in the HS766T cell line promotes chemoresistance to mTOR inhibitor temsirolimus [79]. Additionally, PSCs produced CXCL12-*α* which inhibited gemcitabine mediated apoptosis of pancreatic cancer cells through an IL-6 autocrine loop [74]. Furthermore, an interesting work showed that CXCL12 RNA expression and protein secretion levels were increased when fibroblasts were cocultured with gemcitabine-treated pancreatic cancer cells resistant to gemcitabine. On the other hand, gemcitabine exposure of these cancer cells induced the increase of CXCR4 protein expression. The strengthening of the CXCL12/CXCR4 axis caused enhanced invasive behavior and in vivo tumorigenicity [80]. Unfortunately, it is not clear which isoform the authors investigated, since the RNA expression data were obtained using a TaqMan probe which covers all CXCL12 isoforms. Regarding immunohistochemistry assays, the product identifier of used antibody is not reported. Probably, they investigated the CXCL12 *α*-isoform, but the “anti-SDF-1*α*” word appears only in the Methods section.

In pancreatic cancer, it has been demonstrated that the chemokine CXCL12 could play both protumor and antitumor roles. Several in vitro studies have investigated the CXCL12 roles by administering it to PDAC cell lines. The treatment of Panc-1 and SW-1990 cell lines with CXCL12-*α* isoform showed the upregulation of the matrix metalloproteinase 2 and 9 (MMP-2 and MMP-9). Mechanistically, MMP-2 upregulation was partially mediated by p38 in Panc-1 cells [77]. The observed expression modification of MMP protein family members was also studied in MiaPaCa2 cell lines. The administration of CXCL12 to this cell line promoted proliferation and invasion through the expression of MMP-2, MMP-9, and uPA (urokinase Plasminogen Activator) proteins [70]. However, in this paper, it is not clear which isoform was used since the catalogue number of the CXCL12 recombinant protein is not reported and, at present, the company that supplied this product sells both *α* and *β* isoforms. In vitro studies went further to explore other potential pathways activated upon CXCL12 treatments. In particular, it was shown that CXCL12 administration on Panc-1 cells increased ERK and Akt phosphorylation, enhancing cell proliferation [81]. The treatment with CXCL12 of MiaPaCa2, HPAF, and ASPC1 cell lines also caused Akt and ERK activation and the consequent phosphorylation and destabilization of the NF-*κ*B inhibitory protein, I*κ*B-*α*. This caused the nuclear accumulation of NF-*κ*B, its binding to SHH (Sonic Hedgehog Homolog) gene promoter, and the consequent expression and release of SHH [82]. Unfortunately, it is not declared which CXCL12 isoform was used in the last two papers. However, these mechanisms are worth further investigating. Indeed, SHH has an important role in the tumor microenvironment as shown in hTERT-HPNE cells, a normal pancreatic cell line, where this ligand induced desmoplasia [83].

Interestingly, the administration of CXCL12 in pancreatic cancer cells showed also the activation of signaling pathways mediated by ACKR3, which, therefore, has not only a scavenger role for CXCL12. Indeed, ACKR3 stimulation by CXCL12 in Panc-1 cell lines leads to ERK1/2 phosphorylation through *β*-arrestin-2 without increasing K-Ras activity unlike CXCR4 stimulation [73]. In this experiment, the catalogue number of CXCL12 protein is not reported, so it is not clear which isoform was used.

In PDAC, other molecules connected to the CXCL12/CXCR4/ACKR3 axis have been discovered. For example, in murine models, it was shown that the loss of the tumor suppressor KLF10 (Kruppel-like factor 10) induced metastases in PDAC mouse models through the activation of CXCL12/CXCR4 pathway [84]. In this work, both an antibody detecting all CXCL12 isoforms and the Mouse Cytokine Array able to detect only the *α*-isoform were used. Moreover, in stellate cells obtained from primary pancreatic cancer tissues, the protein Galectin-1 is involved in stimulating the production of CXCL12 through the activation of NF-*κ*B [16]. In this study, different techniques have been applied to investigate different CXCL12 isoforms. In particular, a specific ELISA kit was used for the CXCL12 *α*-isoform; an antibody recognizing all isoforms was used for immunohistochemically staining; RT-qPCR primers detecting both the *α* and *δ* isoforms were used for CXCL12 mRNA quantification.

A recent study proposed an additional mechanism for PDAC neural invasion mediated by CXCL12 [71]. It was already known that cancer cells express chemokine receptors and therefore they are attracted by the chemokines released from the nerves. Through the nerves, cancer cells disseminate and give rise to metastases. Recently, it was shown that precancerous cells release chemokines in order to attract Schwann cells (SC) from the nerve and induce tumor dissemination in early carcinogenesis. In particular, in vitro and in vivo studies in murine models have shown that PDAC cells, by the release of CXCL12-*α*, attract SC cells and determine a decrease in pain sensation given by alteration of SC, spinal astrocytes, and microglia molecular pain pathways [71].

Most of the studies have assigned a protumoral role to CXCL12 in several cancer types, but a study demonstrated that CXCL12 could play also an antitumor role in pancreatic cancer. Indeed, the stable CXCL12 gene reexpression in MiaPaCa2 cancer cells, which is usually epigenetically silenced, caused a significant decrease in tumor growth and migration. In particular, the cell cycle was arrested, and the migration and liver metastases development were reduced. These factors led to tumor growth decrease, both in vivo and in vitro, and to a survival rate increase [76]. Unfortunately, it is unclear whether all the CXCL12 isoforms or only some of them play this antitumor role. Indeed, according to the manufacturer, the antibody used for sandwich ELISA recognizes both CXCL12 *α* and *β* isoforms, but probably its cross-reactivity with the other isoforms was not assessed.

Overall, very little is known about the functional differences among the CXCL12 isoforms, especially in PDAC. The dissection of their specific functions would allow a deeper understanding of the PDAC carcinogenesis and progression mechanisms. Moreover, CXCL12 isoforms could represent new prognostic and predictive biomarkers for several cancers. For example, they could be dosed for a better patient staging, prognosis, and prediction of the metastatic potential and tropism. Regarding the latter, it is known that CXCL12-*γ* levels are associated with metastatic tropism for bone marrow [65]. In preclinical studies and clinical trials, the inhibition of the CXCR4 receptor by, for example, the CXCR4 antagonists AMD3100 and TN14003 and the CXCL12 analogue CTCE-9908, showed promising antitumor effects in different cancers, including pancreatic cancer [40, 85]. Besides anti-CXCL12 antibodies, the only molecule neutralizing all isoforms of CXCL12 is the L-RNA aptamer NOX-A12 [85], which is currently under evaluation in clinical trials as anticancer agent in chronic lymphoblastic leukemia, multiple myeloma, and metastatic colorectal and pancreatic cancer with liver metastasis (source: Clinicaltrials.gov). However, since some molecular differences among CXCL12 isoforms exist, once the specific functions of each isoform in tumor development and progression are clarified, it will be necessary to design CXCL12 isoform-specific therapies.

## 6. Conclusions

In conclusion, data show that CXCL12/CXCR4/ACKR3 axis is involved in keeping the communication between pancreatic cancer and its microenvironment. Most of the results attribute a protumor role to CXCL12, but it could also have an opposite role. Actually, there are six CXCL12 isoforms, and a seventh predicted variant has been identified, so it is possible that they have different effects. Although many studies have investigated the role of this chemokine, they have not clarified the functions of each isoform in pancreatic cancer yet. This is, in part, because it is not always clear which isoform was investigated and, in part, because the recombinant *δ*, *ε*, *θ* isoforms are still not available. Therefore, further studies would be useful to evaluate the specific role of each variant related to pancreatic cancer. The knowledge of these mechanisms could suggest novel strategies to treat PDAC; indeed, it could emerge that some CXCL12 isoforms should be blocked or administered. However, future studies are necessary to establish the optimal stage for this intervention.

## Figures and Tables

**Table 1:** CXCL12 sequence information. The gene CXCL12 (alias SDF1, NCBI Gene ID: 6387) produces 7 splicing isoforms. Data about different CXCL12 sequence variants are reported. Note that, according to NCBI Ref Seq, the transcript variant 5 corresponds to protein isoform 7.

ISOFORM	UNIPROT ID	LENGTH (WEIGHT)	REFSEQ ID
*α*	P48061-2	89AA (10,103 KDa)	NP_954637.1, NM_199168.3
*β*	P48061-1	93AA (10,666 KDa)	NP_000600.1, NM_000609.6
*γ*	P48061-3	119AA (13,705 KDa)	NP_001029058.1, NM_001033886.2
*δ*	P48061-4	140AA (15,495 KDa)	NP_001171605.1, NM_001178134.1
*ε*	P48061-5	90AA (10,192 KDa)	/
*θ*	P48061-6	100AA (11,395 KDa)	/
7	P48061-7	103AA (11,004 KDa)	NP_001264919.1, NM_001277990.1

## References

[1] Deramaudt T., Rustgi A. K. (2005). Mutant KRAS in the initiation of pancreatic cancer. *Biochimica et Biophysica Acta (BBA) - Reviews on Cancer*.

[2] Feldmann G., Beaty R., Hruban R. H., Maitra A. (2007). Molecular genetics of pancreatic intraepithelial neoplasia. *Journal of Hepato-Biliary-Pancreatic Sciences*.

[3] Furukawa T., Sunamura M., Horii A. (2006). Molecular mechanisms of pancreatic carcinogenesis. *Cancer Science*.

[4] Pillarisetty V. G. (2014). The pancreatic cancer microenvironment: An immunologic battleground. *OncoImmunology*.

[5] Carr R. M., Fernandez-Zapico M. E. (2016). Pancreatic cancer microenvironment, to target or not to target?. *EMBO Molecular Medicine*.

[6] Weber C. E., Kuo P. C. (2012). The tumor microenvironment. *Surgical Oncology*.

[7] Daniel S. K., Sullivan K. M., Labadie K. P., Pillarisetty V. G. (2019). Hypoxia as a barrier to immunotherapy in pancreatic adenocarcinoma. *linical and Translational Medicine*.

[8] Pan B., Liao Q., Niu Z., Zhou L., Zhao Y. (2015). Cancer-associated fibroblasts in pancreatic adenocarcinoma. *Future Oncology*.

[9] Uzunparmak B., Sahin I. H. (2019). Pancreatic cancer microenvironment: a current dilemma. *linical and Translational Medicine*.

[10] Awaji M., Singh R. (2019). Cancer-associated fibroblasts’ functional heterogeneity in pancreatic ductal adenocarcinoma. *Cancers*.

[11] Gardian K., Janczewska S., Olszewski W. L., Durlik M. (2012). Analysis of pancreatic cancer microenvironment: Role of macrophage in-filtrates and growth factors expression. *Journal of Cancer*.

[12] Sun Q., Zhang B., Hu Q. (2018). The impact of cancer-associated fibroblasts on major hallmarks of pancreatic cancer. *Theranostics*.

[13] Looi C., Chung F. F., Leong C., Wong S., Rosli R., Mai C. (2019). Therapeutic challenges and current immunomodulatory strategies in targeting the immunosuppressive pancreatic tumor microenvironment. *Journal of Experimental & Clinical Cancer Research*.

[14] Lowenfels A. B., Maisonneuvi P., Cavallini G. (1993). Pancreatitis and the risk of pancreatic cancer. *The New England Journal of Medicine*.

[15] De Veirman K., Rao L., De Bruyne E. (2014). Cancer associated fibroblasts and tumor growth: focus on multiple myeloma. *Cancers*.

[16] Qian D., Lu Z., Xu Q. (2017). Galectin-1-driven upregulation of SDF-1 in pancreatic stellate cells promotes pancreatic cancer metastasis. *Cancer Letters*.

[17] Matsuo Y., Ochi N., Sawai H. (2009). CXCL8/IL-8 and CXCL12/SDF-1alpha co-operatively promote invasiveness and angiogenesis in pancreatic cancer. *International Journal of Cancer*.

[18] Teicher B. A., Fricker S. P. (2010). CXCL12 (SDF-1)/CXCR4 pathway in cancer. *Clinical Cancer Research*.

[19] Ene-Obong A., Clear A. J., Watt J. (2013). Activated pancreatic stellate cells sequester CD8+ T cells to reduce their infiltration of the juxtatumoral compartment of pancreatic ductal adenocarcinoma. *Gastroenterology*.

[20] Feig C., Jones J. O., Kraman M. (2013). Targeting CXCL12 from FAP-expressing carcinoma-associated fibroblasts synergizes with anti-PD-L1 immunotherapy in pancreatic cancer. *Proceedings of the National Acadamy of Sciences of the United States of America*.

[21] Dougan S. K. (2017). The pancreatic cancer microenvironment. *Cancer Journal (United States)*.

[22] Padoan A., Plebani M., Basso D. (2019). Inflammation and pancreatic cancer: focus on metabolism, cytokines, and immunity. *International Journal of Molecular Sciences*.

[23] Pease J. E., Williams T. J. (2006). The attraction of chemokines as a target for specific anti-inflammatory therapy. *British Journal of Pharmacology*.

[24] Stone M., Hayward J., Huang C., E. Huma Z., Sanchez J. (2017). Mechanisms of regulation of the chemokine-receptor network. *International Journal of Molecular Sciences*.

[25] Metzemaekers M., Vanheule V., Janssens R., Struyf S., Proost P. (2018). Overview of the mechanisms that may contribute to the non-redundant activities of interferon-inducible CXC chemokine receptor 3 ligands. *Frontiers in Immunology*.

[26] Strieter R. M., Polverini P. J., Kunkel S. L. (1995). The functional role of the ELR motif in CXC chemokine-mediated angiogenesis. *The Journal of Biological Chemistry*.

[27] Janssens R., Struyf S., Proost P. (2018). The unique structural and functional features of CXCL12. *Cellular & Molecular Immunology*.

[28] Nazari A., Khorramdelazad H., Hassanshahi G. (2017). Biological/pathological functions of the CXCL12/CXCR4/CXCR7 axes in the pathogenesis of bladder cancer. *International Journal of Clinical Oncology*.

[29] Marcuzzi E., Angioni R., Molon B., Calì B. (2019). Chemokines and chemokine receptors: orchestrating tumor metastasization. *International Journal of Molecular Sciences*.

[30] Arimont M., Sun S.-L., Leurs R., Smit M., De Esch I. J. P., De Graaf C. (2017). Structural analysis of chemokine receptor-ligand interactions. *Journal of Medicinal Chemistry*.

[31] Janssens R., Struyf S., Proost P. (2018). Pathological roles of the homeostatic chemokine CXCL12. *Cytokine & Growth Factor Reviews*.

[32] Murdoch C., Finn A. (2000). Chemokine receptors and their role in inflammation and infectious diseases. *Blood*.

[33] Krikun G. (2018). The CXL12/CXCR4/CXCR7 axis in female reproductive tract disease: Review. *American Journal of Reproductive Immunology*.

[34] Teixidó J., Martínez-Moreno M., Díaz-Martínez M., Sevilla-Movilla S. (2018). The good and bad faces of the CXCR4 chemokine receptor. *The International Journal of Biochemistry & Cell Biology*.

[35] Yu L., Cecil J., Peng S.-B. (2006). Identification and expression of novel isoforms of human stromal cell-derived factor 1. *Gene*.

[36] Meng W., Xue S., Chen Y. (2018). The role of CXCL12 in tumor microenvironment. *Gene*.

[37] Janowski M. (2009). Functional diversity of SDF-1 splicing variants. *Cell Adhesion & Migration*.

[38] Guyon A. (2014). CXCL12 chemokine and its receptors as major players in the interactions between immune and nervous systems. *Frontiers in Cellular Neuroscience*.

[39] Zhou Y., Cao H., Li W., Zhao L. (2018). The CXCL12 (SDF-1)/CXCR4 chemokine axis: Oncogenic properties, molecular targeting, and synthetic and natural product CXCR4 inhibitors for cancer therapy. *Chinese Journal of Natural Medicines*.

[40] Zhou W., Guo S., Liu M., Burow M., Wang G. (2017). Targeting CXCL12/CXCR4 axis in tumor immunotherapy. *Current Medicinal Chemistry*.

[41] Pawig L., Klasen C., Weber C., Bernhagen J., Noels H. (2015). Diversity and inter-connections in the CXCR4 chemokine receptor/ligand family: Molecular perspectives. *Frontiers in Immunology*.

[42] Chu T., Shields L. B. E., Zhang Y. P., Feng S.-Q., Shields C. B., Cai J. (2017). CXCL12/CXCR4/CXCR7 chemokine axis in the central nervous system: therapeutic targets for remyelination in demyelinating diseases. *The Neuroscientist*.

[43] De La Luz Sierra M., Yang F, Narazaki M (2004). Differential processing of stromal-derived factor-1alpha and stromal-derived factor-1beta explains functional diversity. *Blood*.

[44] Allami R. H., Graf C., Martchenko K. (2016). Analysis of the expression of SDF-1 splicing variants in human colorectal cancer and normal mucosa tissues. *Oncology Letters*.

[45] Zhao X., Zhu D., Zhang H. (2019). A natural “GA” insertion mutation in the sequence encoding the 3'UTR of CXCL12/SDF-1alpha: Identification, characterization, and functional impact on mRNA splicing. *Gene*.

[46] Zhao S., Chang S. L., Linderman J. J., Feng F. Y., Luker G. D. (2014). A comprehensive analysis of CXCL12 isoforms in breast cancer1,2. *Translational Oncology*.

[47] Santiago B., Calonge E., Rey M. J. D. (2011). CXCL12 gene expression is upregulated by hypoxia and growth arrest but not by inflammatory cytokines in rheumatoid synovial fibroblasts. *Cytokine*.

[48] Burger J. A., Kipps T. J. (2006). CXCR4: a key receptor in the crosstalk between tumor cells and their microenvironment. *Blood*.

[49] Naumann U., Cameroni E., Pruenster M. (2010). CXCR7 functions as a scavenger for CXCL12 and CXCL11. *PLoS ONE*.

[50] Christopherson K. W., Hangoc G., Broxmeyer H. E. (2002). Cell surface peptidase CD26/dipeptidylpeptidase IV regulates CXCL12/stromal cell-derived factor-1*α*-mediated chemotaxis of human cord blood CD34+ progenitor cells. *The Journal of Immunology*.

[51] Lambeir A.-M., Proost P., Durinx C. (2001). Kinetic investigation of chemokine truncation by CD26/dipeptidyl peptidase IV reveals a striking selectivity within the chemokine family. *The Journal of Biological Chemistry*.

[52] McQuibban G. A., Butler G. S., Gong J.-H. (2001). Matrix metalloproteinase activity inactivates the CXC chemokine stromal cell-derived factor-1. *The Journal of Biological Chemistry*.

[53] Delgado M. B., Clark-Lewis I., Loetscher P. (2001). Rapid inactivation of stromal cell-derived factor-1 by cathepsin G associated with lymphocytes. *European Journal of Immunology*.

[54] Valenzuela-Fernández A., Planchenault T., Baleux F. (2002). Leukocyte elastase negatively regulates stromal cell-derived factor-1 (SDF-1)/CXCR4 binding and functions by amino-terminal processing of SDF-1 and CXCR4. *The Journal of Biological Chemistry*.

[55] Scala S. (2015). Molecular pathways: targeting the CXCR4-CXCL12 Axis-Untapped potential in the tumor microenvironment. *Clinical Cancer Research*.

[56] Luker K. E., Steele J. M., Mihalko L. A., Ray P., Luker G. D. (2010). Constitutive and chemokine-dependent internalization and recycling of CXCR7 in breast cancer cells to degrade chemokine ligands. *Oncogene*.

[57] Łuczak M. W., Roszak A., Pawlik P. (2012). Transcriptional analysis of CXCR4, DNMT3A, DNMT3B and DNMT1 gene expression in primary advanced uterine cervical carcinoma. *International Journal of Oncology*.

[58] Gustavsson M., Wang L., van Gils N. (2017). Structural basis of ligand interaction with atypical chemokine receptor 3. *Nature Communications*.

[59] Murphy P. M., Heusinkveld L. (2018). Multisystem multitasking by CXCL12 and its receptors CXCR4 and ACKR3. *Cytokine*.

[60] Balabanian K., Lagane B., Infantino S. (2005). The chemokine SDF-1/CXCL12 binds to and signals through the orphan receptor RDC1 in T lymphocytes. *The Journal of Biological Chemistry*.

[61] Crump M. P., Gong J.-H., Loetscher P. (1997). Solution structure and basis for functional activity of stromal cell-derived factor-1; dissociation of CXCR4 activation from binding and inhibition of HIV-1. *EMBO Journal*.

[62] Spinosa P. C., Luker K. E., Luker G. D., Linderman J. J. (2017). The CXCL12/CXCR7 signaling axis, isoforms, circadian rhythms, and tumor cellular composition dictate gradients in tissue. *PLoS ONE*.

[63] Altenburg J. D., Broxmeyer H. E., Jin Q., Cooper S., Basu S., Alkhatib G. (2007). A naturally occurring splice variant of CXCL12/stromal cell-derived factor 1 is a potent human immunodeficiency virus type 1 inhibitor with weak chemotaxis and cell survival activities. *Journal of Virology*.

[64] Connell B. J., Sadir R., Baleux F. (2016). Heparan sulfate differentially controls CXCL12alpha- and CXCL12gamma-mediated cell migration through differential presentation to their receptor CXCR4. *Science Signaling*.

[65] Ray P., Stacer A. C., Fenner J. (2014). CXCL12-*γ* in primary tumors drives breast cancer metastasis. *Oncogene*.

[66] Gosalbez M., Hupe M. C., Lokeshwar S. D. (2014). Differential expression of SDF-1 isoforms in bladder cancer. *The Journal of Urology*.

[67] Jung Y., Cackowski F. C., Yumoto K. (2018). CXCL12*γ* promotes metastatic castration-resistant prostate cancer by inducing cancer stem cell and neuroendocrine phenotypes. *Cancer Research*.

[68] Zhong W., Chen W., Zhang D. (2012). CXCL12/CXCR4 axis plays pivotal roles in the organ-specific metastasis of pancreatic adenocarcinoma: a clinical study. *Experimental and Therapeutic Medicine*.

[69] Liu C., Pham K., Luo D. (2013). Expression and functional heterogeneity of chemokine receptors CXCR4 and CXCR7 in primary patient-derived glioblastoma cells. *PLoS ONE*.

[70] Shen B., Zheng M.-Q., Lu J.-W., Jiang Q., Wang T.-H., Huang X.-E. (2013). CXCL12-CXCR4 promotes proliferation and invasion of pancreatic cancer cells. *Asian Pacific Journal of Cancer Prevention*.

[71] Demir I. E., Kujundzic K., Pfitzinger P. L. (2017). Early pancreatic cancer lesions suppress pain through CXCL12-mediated chemoattraction of Schwann cells. *Proceedings of the National Acadamy of Sciences of the United States of America*.

[72] Zhang J., Liu C., Mo X., Shi H., Li S. (2018). Mechanisms by which CXCR4/CXCL12 cause metastatic behavior in pancreatic cancer. *Oncology Letters*.

[73] Heinrich E. L., Lee W., Lu J., Lowy A. M., Kim J. (2012). Chemokine CXCL12 activates dual CXCR4 and CXCR7-mediated signaling pathways in pancreatic cancer cells. *Journal of Translational Medicine*.

[74] Zhang H., Wu H., Guan J. (2015). Paracrine SDF-1*α* signaling mediates the effects of PSCs on GEM chemoresistance through an IL-6 autocrine loop in pancreatic cancer cells. *Oncotarget *.

[75] Gao Z., Wang X., Wu K., Zhao Y., Hu G. (2010). Pancreatic stellate cells increase the invasion of human pancreatic cancer cells through the stromal cell-derived factor-1/CXCR4 Axis. *Pancreatology*.

[76] Roy I., Zimmerman N. P., Mackinnon A. C., Tsai S., Evans D. B., Dwinell M. B. (2014). CXCL12 chemokine expression suppresses human pancreatic cancer growth and metastasis. *PLoS ONE*.

[77] Pan F., Ma S., Cao W. (2013). SDF-1*α* upregulation of MMP-2 is mediated by p38 MAPK signaling in pancreatic cancer cell lines. *Molecular Biology Reports*.

[78] Liu Z., Teng X., Meng X., Wang B. (2014). Expression of stromal cell-derived factor 1 and CXCR7 ligand receptor system in pancreatic adenocarcinoma. *World Journal of Surgical Oncology*.

[79] Weekes C. D., Song D., Arcaroli J. (2012). Stromal cell-derived factor 1alpha mediates resistance to mTOR-directed therapy in pancreatic cancer. *Neoplasia*.

[80] Morimoto M., Matsuo Y., Koide S. (2016). Enhancement of the CXCL12/CXCR4 axis due to acquisition of gemcitabine resistance in pancreatic cancer: Effect of CXCR4 antagonists. *BMC Cancer*.

[81] Shen X., Artinyan A., Jackson D., Thomas R. M., Lowy A. M., Kim J. (2010). Chemokine receptor CXCR4 enhances proliferation in pancreatic cancer cells through akt and erk dependent pathways. *Pancreas*.

[82] Singh A. P., Arora S., Bhardwaj A. (2012). CXCL12/CXCR4 protein signaling axis induces sonic hedgehog expression in pancreatic cancer cells via extracellular regulated kinase- and Akt kinase-mediated activation of nuclear factor kappaB: implications for bidirectional tumor-stromal interactions. *The Journal of Biological Chemistry*.

[83] Bailey J. M., Swanson B. J., Hamada T. (2008). Sonic hedgehog promotes desmoplasia in pancreatic cancer. *Clinical Cancer Research*.

[84] Weng C.-C., Hawse J. R., Subramaniam M. (2017). KLF10 loss in the pancreas provokes activation of SDF-1 and induces distant metastases of pancreatic ductal adenocarcinoma in the KrasG12D p53flox/flox model. *Oncogene*.

[85] Barbieri F., Bajetto A., Thellung S., Würth R., Florio T. (2016). Drug design strategies focusing on the CXCR4/CXCR7/CXCL12 pathway in leukemia and lymphoma. *Expert Opinion on Drug Discovery*.

